# The impact of proprioception impairment on gait function in stroke survivors: a comprehensive review

**DOI:** 10.3389/fneur.2025.1577919

**Published:** 2025-05-12

**Authors:** Maciej Kochman, Marta Kasprzak, Aleksandra Kielar

**Affiliations:** ^1^Department of Clinical Physiotherapy in Musculoskeletal Disorders, Institute of Physiotherapy, Faculty of Health Sciences and Psychology, Collegium Medicum, University of Rzeszów, Rzeszów, Poland; ^2^Faculty of Medicine, Collegium Medicum, University of Rzeszów, Rzeszów, Poland

**Keywords:** stroke, rehabilitation, physiotherapy, gait disorders, gait analysis, somatosensory disorders, proprioception, position sense

## Abstract

Stroke survivors often experience sensory, cognitive, and motor consequences with gait disorders as a common problem. Therefore, there is a need for a deeper understanding of how neurological deficits affect the functioning of patients after a stroke. Current scientific literature lacks research on proprioception impairment, and gait recovery after stroke. In this narrative review, we discussed and summarized the current knowledge about the abnormal post-stroke gait pattern, the role of proprioception in motor control, methods of proprioception assessment, and the association between abnormal gait and proprioception deficit in stroke survivors. The present findings must be interpreted with some caution as current evidence is limited, as well as the correlation does not imply causation and might be underestimated by attributes of current tests for proprioception and motor function. Further studies are needed to better explain the mechanisms behind proprioception deficits and their association with functional recovery, as well as to investigate the cause-effect relationship.

## 1 Introduction

Stroke is a serious health problem exploiting a significant proportion of health care system budgets worldwide (1). The World Health Organization (WHO) indicates that stroke is the incoming epidemic of the 21st century and a further increase in stroke rates is expected worldwide, especially in younger patients (2). According to the World Stroke Organization (WSO), stroke remains the second most common cause of death and the third most common cause of combined death and disability expressed by disability-adjusted life-years lost (DALYs) worldwide (3). The definition of stroke includes rapidly developing clinical symptoms of focal or global disturbance of cerebral function, while signs last at least 24 h or lead to death and their cause is no other than vascular origin (4).

Despite a decline in stroke mortality rates, the prevalence of people living with stroke consequences has risen due to an increasing and aging population. This leads to a greater need for long-term rehabilitation (5). People after a stroke often experience sensory, cognitive, and motor consequences (6). Gait disorders are a common problem in stroke survivors as they constitute one of the main functional limitations that affect the quality of life and increase the risk of falls. Moreover, independent walking is an important factor in overall health and one of the fundamental goals of stroke rehabilitation (7). Stroke survivors may have different gait patterns depending on the variety of sensorimotor disorders (8). However, the relationship between sensory impairment and other impairments or functional deficits following a stroke is unclear and has not been widely explored in the literature. Moreover, despite the expectation that sensory or motor deficits may be associated with stroke severity due to lesion size and location, the association between these impairments is still not well understood (9). Previous studies showed that lesions in the supramarginal gyrus, arcuate fasciculus, and Heschl’s gyrus are linked to poor proprioceptive recovery. Also, proprioception impairment is common and persistent after stroke, particularly in the cortical and subcortical lesions (10).

Due to the constantly increasing incidence of stroke and the increasing number of people living with its consequences, there is a need for a deeper understanding of how neurological deficits, including proprioception deficit, affect the functioning of patients after a stroke. As this topic is underexplored in the scientific literature, we were prompted to perform this narrative literature review, which, according to the best knowledge of authors, is the very first review providing a comprehensive understanding of the role of proprioception in post-stroke gait recovery. In this review, we present and summarize the current knowledge about the abnormal post-stroke gait pattern, role of proprioception in motor control, methods of proprioception assessment, and the association between abnormal gait and proprioception deficit in stroke survivors.

## 2 Hemiplegic gait and its characteristics

People after a stroke usually present various neurological deficits, such as motor, sensory, cognitive, or perceptual impairments. One of the major post-stroke disorders is a motor deficit manifesting in contralateral hemiparesis to the cerebrovascular incident, which decreases the capacity of affected limbs to initiate and control movements and maintain balance, resulting in abnormal gait patterns (11). Abnormal gait is very common after stroke as more than 80% of stroke survivors suffer from varying degrees of gait abnormalities, and about 25% of them have an enduring impairment requiring full physical assistance, despite long-term rehabilitation (12).

A characteristic gait pattern in stroke patients is the so-called hemiparetic (or hemiplegic) gait. This gait pattern is characterized by specific temporal and spatial patterns, including reduced cadence, reduced walking speed, increased step width, increased duration of the double stance phase, and asymmetric loading of a single lower limb. After a stroke, the affected limb shows a prolonged duration of the swing phase, caused by a deficit of the force required to move forward, and a shortened duration of the stance phase. As a result, a shortened duration of the swing phase and an increased duration of the stance phase are observed in the non-affected lower limb (11, 13, 14).

Moreover, hemiplegic gait is also characterized by asymmetry and shortened step length of the non-affected lower limb, compared to the gait of healthy individuals (14, 15). Also, the kinematic parameters of hemiparetic gait show increased movement of the trunk in the lateral and sagittal planes, greater movement of the upper part of the trunk in the transverse plane, reduced interphase rotation of the upper and lower parts of the trunk, and reduced stability and symmetry compared to non-pathological gait. Unlike the limbs, the functioning of the trunk after a stroke is impaired bilaterally. Both the part of the trunk on the paretic side and the part of the trunk on the side indirectly affected after a stroke are characterized by a reduced level of activity and reduced synchronization of its muscle work (14, 16).

Muscle strength deficits in the trunk muscles affect its balance and coordination and cause changes in the biomechanics of gait. Studies indicate that weakening of the trunk extensors and flexors and associated balance disorders contribute to reduced functional independence in walking and transfers getting up from a sitting position, as well as gait speed (17). Also, walking speed is mainly influenced by the weakening of the hip flexors and knee extensors, while gait symmetry is significantly influenced primarily by the degree of spasticity of the ankle flexors, however, the ankle flexors also affect walking speed (18–20).

Foot clearance is an important gait parameter that affects tripping during the swing phase, which is a serious cause of falls. In healthy subjects, foot clearance is determined by hip and knee joint flexion and ankle dorsiflexion (21, 22). In patients with hemiplegic gait, during the swing phase, flexion movement of the lower limb is impaired distinguishing “foot drop” and “stiff-knee gait” among them, which cause characteristic movements such as hip hiking and circumduction (21, 23, 24). These compensatory movements are performed to achieve better foot clearance to compensate for the reduced lower limb flexion (25). Foot drop is one of the most common post-stroke disorders affecting the gait of people after stroke, which is associated with the weakening or lack of voluntary control of the ankle dorsal flexors and/or increased spasticity of the plantar flexors (26–28). It disturbs the dorsal flexion of the foot during the swing phase and causes disorders in accepting and transferring body weight in the initial foot contact and the stance phases (27). Approximately 60% of stroke survivors with gait impairment experience stiff-knee gait, also known as stiff-legged gait. The definition of this gait pattern in the literature varies depending on the source (29). However, it is characterized by reduced knee flexion during the swing phase of the gait cycle (12, 29, 30).

## 3 Proprioception and its role in motor control

Proprioception is the sensation of body position and movement and it is essential for movement control as it provides inputs to internal models that link sensory signals with motor commands (31, 32). It involves signals from mechanoreceptors (transducers that transform mechanical stimuli into action potentials) positioned in muscles, tendons, and joint capsules (proprioceptors), whereas information received from cutaneous mechanoreceptors (cutaneous stretch receptors) related to tactile sensations, is regarded as additional sensory sources completing proprioceptive inputs (31). Muscle proprioceptors include muscle spindles, which respond to the stretching of muscle fibers, and Golgi tendon organs. Pacinian and Ruffini corpuscles are considered joint receptors because of their strong association with ligaments, but they can be observed throughout the body. Skin proprioception is also associated with the same group of sensory receptors. However, they are embedded in the skin and deep connective tissues covering mobile joints or muscles. Sensory neurons, including Meissner and Merkel cells, are activated by movement or touch on the skin (33).

All collected proprioceptive information is processed in the spinal cord, brain stem, higher cortical centers and subcortical cerebral nuclei, and cerebellum. This data is then used in daily activities, exercises, and sports (34) and it allows one to maintain posture, maneuver in the dark, and manipulate objects out of sight. Moreover, in people with correct proprioception, eliminating visual information still allows them to determine the position and movement of individual body parts (35). It has also been shown that information from both the joint and the skin is related to the sense of upright position, which affects balance. This also plays a critical role in providing feedback on the distribution of body weight on individual limbs (35, 36). Proprioception disorders can occur as a result of improper functioning of the musculoskeletal system due to injury, aging, or neurological disorders such as stroke (36, 37).

## 4 Proprioception assessment and its impact on gait

**Figure 1:**
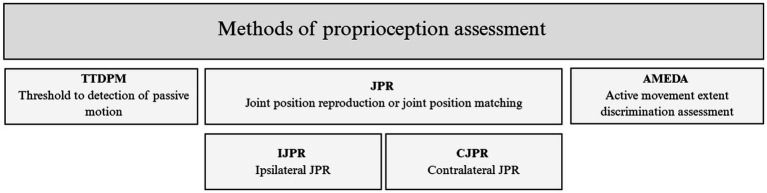
Methods of proprioception assessment.

Due to the crucial role of proprioception in the human body, it is important to use specific methods and tools to assess it. Various techniques have been introduced in the literature to investigate proprioceptive mechanisms. Three primary methods for evaluating proprioception are: (1) TTDPM (threshold to detection of passive motion), (2) JPR (joint position reproduction or joint position matching), and (3) AMEDA (active movement extent discrimination assessment) (38) (Figure 1).

A standard TTDPM assessment includes indicating the first detection of passive motion at the joint by a blindfolded individual. This outcome is measured by the degree of the joint movement or the time elapsed before the individual indicates the detection of movement. Another variable is the detection of the movement direction of the examined body part (e.g., flexion or extension) (39).

In the JPR assessment, a blindfolded individual is asked to recreate a position of a joint angle that they previously experienced using their ipsilateral or the contralateral limb. The proprioceptive sensitivity is indicated by the magnitude of matching errors, as it is believed that subjects making significant position-matching errors are somehow proprioceptively deficient (40). Three methods of JPR assessment have been described in the literature: IJPR (ipsilateral JPR) and two CJPR (contralateral) assessments. In the IJPR assessment, the target position of the joint is passively or actively introduced to the individual for a few seconds prior. After that, the examined limb returns passively or actively to the initial start position. Then the participant is asked to recreate the target position of the joint either by pressing the stop button when the same limb is being moved passively or by moving the joint to the target position if the motion reproduction is active. In CJPR assessments, one method is identical to the JPR except for using the contralateral limb when recreating the target position. In the second CJPR assessment, instead of returning to the start position, the examined limb remains in the target position and the contralateral limb is required to recreate that position (38, 41).

Unlike the previous methods, the AMEDA assessment is performed in a standing position in an unconstrained weight-bearing stance that mimics the conditions that proprioception would encounter outside the laboratory in daily life (42). This method indicates an individual’s somatosensory function by assessing their sensitivity to detect small differences in the angular position of ankle inversion (43). In this method, the participants are asked to indicate in absolute terms the angle at which the plate is displaced from the horizontal (44). This approach provides ankle movement discrimination scores obtained with a single stimulus that utilizes a fixed set of stimuli with one variable stimulus value presented on each trial (45).

Although the AMEDA assessment demonstrates good reliability in healthy adults, it may be impacted by various factors such as chronic ankle instability (46), lapses in attention, or disengagement from the task (47). Despite its established utility for discriminating between participant groups, concerns have been raised about using the AMEDA to classify proprioception acuity at an individual level. Issues included poor test–retest reliability, a small number of stimuli, task difficulty, and a lack of sequencing effects in the analysis (44). According to Waddington and Witchalls, better reliability in AMEDA assessments is achieved with a shorter, 25-response protocol rather than more traditional 50-response protocol, as the shorter version prevents disengagement or inattention (47). Additionally, as noted by Krewer et al. (48), AMEDA measurements do not indicate the proprioception level of a specific joint. Instead, they represent a “multi-modal, multi-joint measure of a multi-segment posture” (48).

To better understand and evaluate the mechanism of proprioception in stroke survivors’ rehabilitation it is essential to use accurate and sensitive assessments and tools. In clinical settings, proprioception assessments were traditionally performed on simple subjective observation-based tests. Usually, the examiner moves the patient’s finger or toe while patients with eyes closed were asked to report the position of this body part. These tests, however, present poor reliability, lack resolution, and display “ceiling effects.” The above-outlined challenges prompted some research groups to design standardized and reliable clinician-administered questionnaires such as the Nottingham Sensory Assessment or Rivermead Assessment of Somatosensory Performance (RASP) (49). Moreover, recently automated and instrumented measurement tools such as electronic goniometers, smartphones, and robot-assisted technologies, have been introduced to assess proprioception impairment (49, 50). Robot-assisted proprioception assessment allows to generation of a rich dataset of kinematic data quantifying impairments that may be difficult to assess by clinical observation-based tests. This large volume of data may be useful in predicting outcomes and planning physiotherapy programs in many conditions, such as stroke (49, 51).

Somatosensory impairment, its nature, and its extent after stroke are not yet fully understood. It is known that it is common after stroke with the prevalence varying from 11 to 60% depending on the heterogeneity of populations, the number of somatosensory modalities, and body parts assessed (52, 53). Among stroke survivors with somatosensory impairment, about 34 to 64% of them suffer from proprioception impairment.

Stroke severity is the main factor affecting somatosensory impairment and initial somatosensory impairment is a great predictive factor for recovery. Moreover, the somatosensory assessment provides useful prognostic information for the functional status of patients. Despite that, the scientific literature lacks research on somatosensory, especially proprioception, impairment, and recovery after stroke (52). On the other hand, the results of present studies indicate the controversies on the impact of sensory function on gait performance, which have been partly linked to the various methods used in assessing sensory function (54). Another reason is that the neuroanatomical injury caused by stroke varies greatly in both location and severity (55). Unfortunately, the understanding of the brain regions involved in processing and lateralization of the proprioceptive signals is still not well explored. It is known that each limb is controlled by contralateral regions of the brain (56). However, some studies suggest that brain activity related to proprioceptive tasks is lateralized, with the right hemisphere being more dominant, regardless of limb dominance (57). Although there is some evidence supporting this lateralization, it is limited and restricted to the upper limbs. For the lower limbs there is some evidence that the non-dominant side is preferred for position-matching tasks. However, contradictory findings regarding the impact of side dominance in lower limb position matching create uncertainty in this area of research (56).

Moreover, after stroke, somatosensory structures may be impacted at some times or preserved at others. Also, proprioception assessment depends on cognitive abilities such as attention and working memory, which are frequently affected in stroke survivors and confounded by fatigue leading to further proprioception assessment variability (55).

A meta-analysis proved a correlation between proprioceptive impairment and motor deficits after stroke. The subgroup analysis revealed multiple factors with positive contributions to this relationship such as proprioception assessed in the axial segment under weight-bearing conditions, proprioception assessed by ipsilateral matching task, and motor function analyzed within ICF domains including movement function, activity independence, and activity performance (58). It was also proved that touch and proprioception are intimately integrated and their impairment affects functional activity. This may suggest that instead of individual sensory retraining, functional task training of both of these impairments may be a more effective treatment (59).

It was observed that in the acute phase of stroke rehabilitation, somatosensory impairment was related to lower functional status, poorer rehabilitation outcomes, and longer length of hospital stay (53). Another study proved that the interaction of knee extensor strength and proprioception on the affected side is strongly linked to gait independence in subacute stroke patients (60).

**Figure 2:**
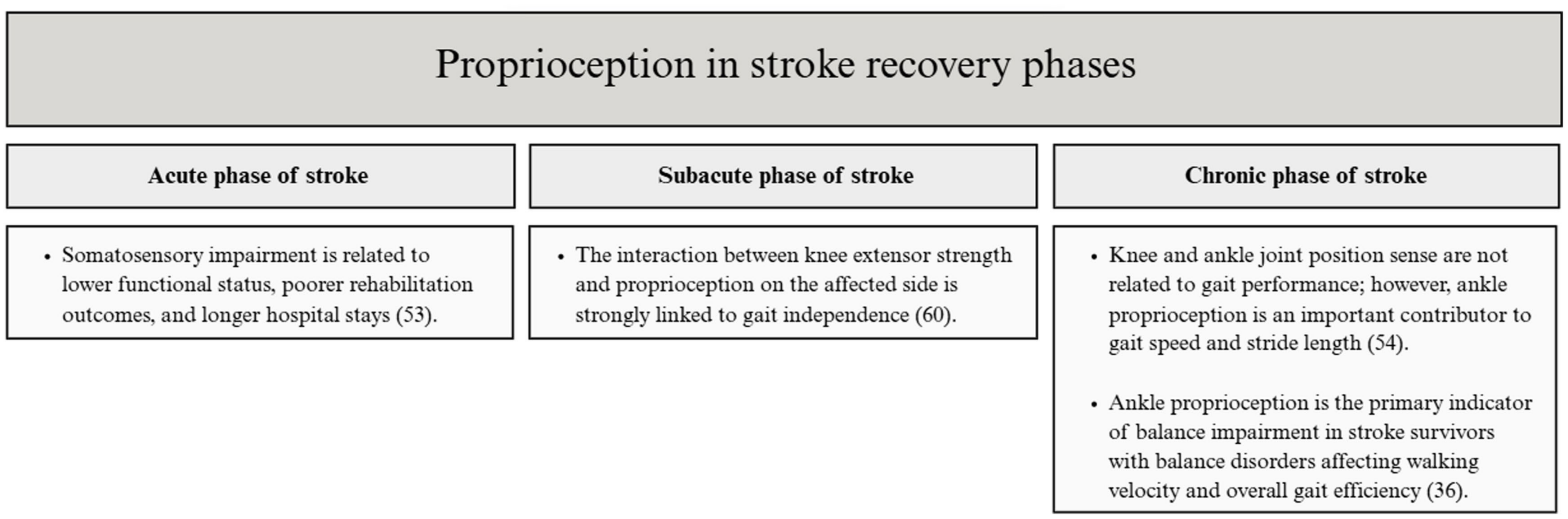
The relationship between proprioception deficit and gait function after stroke.

In the chronic phase of stroke rehabilitation, knee and ankle joint position sense is not related to gait performance, however, ankle proprioception was an important contributor to gait speed and stride length, which supports the importance of proprioception in the decision-making process about changing the gait pattern in stroke survivors (54). Another study also showed that ankle proprioception is the main indicator of balance impairment in stroke survivors with balance disorders in the chronic recovery stage affecting walking velocity and overall gait efficiency. This may demonstrate that ankle proprioception should be included in post-stroke lower limb rehabilitation (36). The relationship between proprioception deficit and gait function in a specific stroke recovery phase has been presented in Figure 2.

Interestingly, proprioception deficit was also observed on the unaffected leg in chronic stroke patients which suggests a possible contribution of peripheral mechanisms (61).

## 5 Summary and future directions

The current understanding of proprioception and its impact on gait function in individuals after stroke remains limited. The present findings should be interpreted with caution, as correlation does not imply causation and may be confounded by limitations inherent in current assessments of proprioception and motor function.

Additional research is necessary to elucidate the cause-and-effect relationships involved and to clarify the mechanisms underlying proprioceptive deficits and their association with functional recovery.

In particular, future research should aim to deepen our understanding of the neuroanatomical foundations of proprioception, including the involvement of specific cortical centers in each cerebral hemisphere. Further research should also investigate whether similar right hemisphere lateralization and non-dominant side preference for proprioception are evident among individuals with left-limb or mixed-limb dominance. Advances in neuroimaging and neurophysiological techniques could provide valuable insights into how various brain regions contribute to proprioceptive processing and recovery following stroke.

Furthermore, targeting both proprioceptive and motor impairments simultaneously may offer a more effective approach to post-stroke rehabilitation. The integration of robotic technologies holds promise for the development of reliable and sensitive proprioceptive assessment tools. Developing and implementing more standardized, reproducible, and accurate devices is essential to quantify proprioceptive function and monitor changes over time. Such advancements would improve diagnostic precision and consequently enable more personalized and targeted rehabilitation strategies.
